# EdgeDenseCalib: Targetless Camera–LiDAR Calibration via Enhanced Edge Feature Densification

**DOI:** 10.3390/s26092690

**Published:** 2026-04-26

**Authors:** Zhiyu He, Zhiwei Cao, Ning Xu, Zhipeng Zhao, Junyi Zhao, Zhao Sheng, Xiaoyu Zhao

**Affiliations:** 1 Signal and Communication Research Institute, China Academy of Railway Sciences Corporation Limited, Beijing 100081, China; hezhiyu@rails.cn (Z.H.); jsxuning@aliyun.com (N.X.); zhaocars@163.com (Z.Z.); 19111070@bjtu.edu.cn (J.Z.); 18810387926@163.com (Z.S.); zhaoxiaoyu059@126.com (X.Z.); 2 State Key Laboratory of Advanced Rail Autonomous Operation, Beijing Jiaotong University, Beijing 100044, China

**Keywords:** camera–LiDAR calibration, sensor fusion, extrinsic parameters, edge feature enhancement, depth discontinuity

## Abstract

Accurate camera–LiDAR calibration is a fundamental prerequisite for reliable perception in autonomous systems. However, traditional methods typically rely on manual intervention or specific calibration targets, which restrict their flexibility and scalability in dynamic, real-world environments. To address the challenge of targetless calibration, we propose EdgeDenseCalib, a novel approach driven by enhanced edge feature densification. A key innovation lies in a two-stage process designed to densify the inherently sparse edge features in LiDAR data, thereby making them highly comparable to the fine-grained edges present in images. Consequently, this facilitates more reliable feature matching between the two cross-modal data sources. An optimization algorithm is subsequently employed to refine the alignment and minimize the reprojection error. Experiments on the KITTI dataset show our method achieves accurate calibration results of 0.105° in mean rotation error and 0.903 cm in mean translation error. Compared to state-of-the-art edge-based methods, our approach significantly improves the rotation accuracy by 33.1% to 89.9%. This work provides a practical and automatic calibration solution, contributing to the development of more robust perception systems for autonomous applications.

## 1. Introduction

The real-time perception of surrounding environment plays an important role in a wide range of applications, including robotics, autonomous driving, and industrial processing control. Accurate environmental understanding is essential for tasks such as obstacle detection, semantic segmentation, and scene reconstruction, which are critical for ensuring safety, efficiency, and reliability in autonomous systems. To achieve comprehensive perception, multi-sensor fusion has become an increasingly popular approach, with cameras and light detection and ranging (LiDAR) sensors being two of the most commonly employed devices [1,2]. Cameras offer rich visual information, including detailed textures, colors, and semantic cues, enabling nuanced scene interpretation but lack of accurate depth information. In contrast, LiDAR provides precise three-dimensional spatial measurements against varying lighting conditions but with sparse point cloud data. The integration of camera and LiDAR data can significantly enhance the performance of environmental perception by leveraging the complementary strengths of both sensors. Thus, to better align the characteristics extracted from camera and LiDAR, the accurate extrinsic calibration is a crucial prerequisite.

In this paper, extrinsic calibration is the estimation procedure for six degrees-of-freedom (6-DoF) transformation between camera and LiDAR coordinate systems. Traditionally, the previous studies on solving the camera–LiDAR extrinsic calibration problem mainly depend on artificial markers, especially checkboard [3,4,5,6,7]. However, those methods are typically applied for offline calibration, making it difficult to accommodate pose deviations or position offsets during device operation, and the process always requires human participation, leading to human-induced errors. In such scenarios, offline calibration techniques cannot provide timely adjustments to extrinsic parameters jitter. Moreover, the use of checkboards necessitates the pre-fabrication of customized calibration targets and the design of specific calibration procedures, which are time-consuming, labor-intensive, and costly [8]. Therefore, although target-based methods yield highly accurate extrinsic calibration results, target-free approaches are more suitable for long-term deployment of sensing devices in real-world environments.

Targetless camera–LiDAR extrinsic calibration breaks through the limitation of target-based methods. Such methods leverage features from natural scenes by extracting typical edges like points, lines, and corners from both 2D images and 3D point cloud data to seek feature correspondences [9,10,11]. The edge features extracted from images contain an abundance of detailed information, which can inadvertently introduce interference in the camera–LiDAR calibration process. Conversely, the edge features obtained from LiDAR are often insufficient. A discrepancy exists between 2D and 3D features due to an imbalance in feature density from both sensors. This situation may lead to ambiguities and increase the complexity of accurately aligning cross-modal data. Moreover, because LiDARs usually scan horizontally, many extracted 2D lines cannot be paired with corresponding 3D features due to poor vertical characteristics [12].

**Figure 1:**
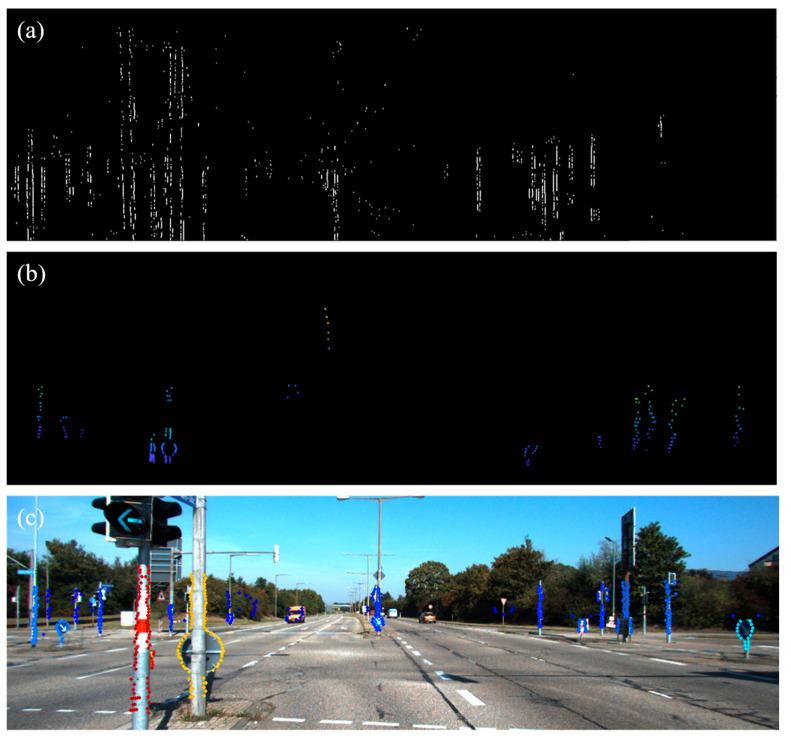
Edge features extracted from an image (**a**) and a point cloud (**b**) from the same scenario. After optimizing the 2D and 3D feature correspondences, the projected point cloud data (**c**) is well aligned with the edge features in the image.

To deal with the aforementioned problem, we propose the EdgeDenseCalib, which makes full use of the 2D and 3D edge features to achieve robust correspondences in various scenarios. For 2D images, we apply Gaussian filtering to suppress noise and a gradient-based method combined with angular constraints to extract prominent linear edges. These orientation constraints help to filter out irrelevant edges and retain those that are most informative for calibration. Moreover, for the 3D point cloud data, we propose an edge extraction method combined with depth discontinuities and a two-stage LiDAR edge-enhancement mechanism to enrich edge expression. Figure 1 illustrates the edge feature extraction result of our method.

A fundamental distinction between our proposed EdgeDenseCalib and existing edge-based methods lies in the treatment of occlusion boundaries. Conventional methods primarily rely on basic depth gradients or intensity variations for LiDAR edge extraction. Consequently, they often suffer from severe feature fragmentation and sparsity, especially at the boundaries between foreground objects and unreturned backgrounds. Our approach explicitly addresses this density imbalance. By combining the CSW module with a novel BPSW module that uniquely utilizes localized missing data as a physical cue, our method captures critical boundary-transition edges that previous methods systematically miss. This dual-stage densification fundamentally ensures a more robust and balanced cross-modal feature alignment.

Our contributions can be summarized as follows:

(1) We propose the EdgeDenseCalib that operates without manual intervention or artificial markers. By leveraging common vertical structures and enhancing point-cloud representations, our approach notably improves edge-feature quality across both sensing modalities.
(2) We introduce a two-stage LiDAR edge-enhancement strategy consisting of a cross-searching window (CSW) module and a background-preserving searching window (BPSW) module. This design enriches sparse point-cloud edges and effectively balances the feature density between images and LiDAR data, enabling more reliable cross-modal matching in natural scenes.
(3) We develop a neighborhood-search optimization method based on the iterative closest point (ICP) framework. It minimizes reprojection errors by aligning LiDAR-projected points with nearby image edges. Extensive evaluation on the KITTI dataset under real-world conditions demonstrates the method’s accuracy and practical viability.

The rest of this paper is organized as follows: Section 2 summarizes related works; Section 3 introduces the EdgeDenseCalib in this paper; Section 4 evaluates the performance of the EdgeDenseCalib in a natural environment; and Section 5 summarizes our research.

## 2. Related Works

The camera–LiDAR extrinsic calibration approaches can be divided into two branches: (1) target-based methods and (2) targetless-based methods, depending on whether a preset calibration target is needed.

### 2.1. Target-Based Methods

Target-based methods usually utilize explicit and predesigned objects, like a chessboard [3,4,5,6], ArUco label [7,13], triangular target [14], or cube target [15,16] for extrinsic matrix estimation. Zhang et al. [17] first introduced a checkboard to solve the extrinsic parameters calibration problem between a two-dimensional LiDAR and a camera. This method optimized the re-projection error by using the points constrained by planes. Unnikrishnan et al. [18] proposed a fast camera–LiDAR calibration toolkit by manually selecting corresponding points from both sensors. Pandey et al. [3] described a calibration method from a minimum of three views. Dhall et al. [19] utilized a rectangle carved checkboard to calibrate extrinsic parameters through selected points. The methods mentioned above always require human intervention through the manual annotation of points from images and point clouds, which introduces manual errors, and the chosen points may not align with each other precisely.

To break through the limitations above, some researchers seek different ways to calculate the camera–LiDAR extrinsic parameters automatically and more intelligently. Liao et al. [20] employed a polygon board to extract the target polygon from the camera and LiDAR. Then, the 2D and 3D features are matched automatically under point-to-line and point-inside-polygon constraints. Huang et al. [6] proposed a corner detection method from point clouds, and connected the edge points to fit the checkboard plane by RANdom SAmple Consensus (RANSAC) algorithm. Cui et al. [21] proposed a temporal-spatial-based geometric feature refinement method to extract effective features from LiDAR, and then estimated 3D points with the reflectance distribution approach to match 2D points using a Perspective-n-Perspective (PnP) solution. However, the target-based methods cannot avoid the existence of a specific target which is limited to laboratory settings. Additionally, such methods are not suitable for frequently adjusting the camera–LiDAR extrinsic parameters in a dynamic environment like automatic driving scenarios.

### 2.2. Targetless Methods

In real-world applications, online estimation and correction of camera–LiDAR extrinsic parameters are the prerequisite for cross-modal data fusion. Targetless methods utilize the ubiquitous presence of natural objects such as tree trunks, utility poles, street lamps, vehicles, and traffic signs by extracting edge features to achieve extrinsic parameter alignment, thereby enabling calibration without the need for predefined targets. Borer et al. [22], Jiang et al. [23], and Liu et al. [24] proposed an automatic extrinsic calibration method by maximizing mutual information from surface intensity or edge features between the sensors. Zhou et al. [25], Xuan et al. [26], and Ishikawa et al. [27] presented hand–eye calibration approaches to estimate extrinsic parameters of a camera–LiDAR system, which only require motion information. Huang et al. [28] developed a motion-based calibration method applying the Gauss–Helmert estimation paradigm, together with a formulation for multi-sensor motion constraint. However, motion-based methods often suffer from limited extrinsic calibration accuracy due to errors in pose estimation. Thus, they are typically employed only for coarse initial estimation [29,30]. Some researchers developed camera–LiDAR extrinsic calibration through a learning-based solution, which is not sensitive to specific features and can learn extrinsic parameters directly from a deep neural network [31,32]. Schneider et al. [33] first introduced a deep learning method, RegNet, for camera–LiDAR extrinsic calibration, extracting features using residual blocks from both sensors. In ref. [34], the authors proposed a LiDAR and camera self-calibration method (LCCNet) using a cost volume network to represent the relationship between images and deep images from point clouds. In ref. [35], CFNet is designed by combining deep learning and geometry methods. The EPnP algorithm within the RANSAC scheme is applied to estimate the extrinsic parameters with 2D–3D correspondences constructed by the calibration flow. Despite their advantages, learning-based methods still face several limitations, including large-scale training cost, unknown generalization capability, and a lack of interpretability.

Many researchers and scholars are devoted to feature-based approaches due to their wide existence in the natural environment. Levison et al. [36] introduced a two-staged online calibration method between camera and LiDAR. They applied depth discontinuities from LiDAR to extract edge features aligning with image edges. However, this method is limited to the calibration scenes, which contain specific edges. Yu et al. [37] decouples the rotation calculation and translation estimation to simultaneously estimate the 2D–3D line correspondences and camera poses in structural environments. Zhang et al. [38] utilized a normal distribution transform (NDT) method combining the current and previous two frames to enhance line features from LiDAR. They established a cost function by re-projecting LiDAR points onto images focusing on both horizontal and vertical features. Ye et al. [39] combined depth-continuous edges, depth-discontinuous edges, and intensity-discontinuous edges to strengthen edge presentation in LiDAR data for cross-modal alignment.

Recent advancements, including EdgeCalib and various multi-feature alignment frameworks, have significantly improved calibration accuracy by leveraging geometric contours and multi-metric optimizations. However, these approaches fundamentally treat 3D edge extraction as a standard gradient-based operation. This paradigm inevitably leads to sparse and discontinuous edge representations on the LiDAR side, failing to fully resolve the inherent density imbalance when matched against high-resolution camera edges. In contrast, our EdgeDenseCalib explicitly targets the densification bottleneck. Rather than solely relying on continuous depth discontinuities, our approach innovatively incorporates the physical limitations of LiDAR scanning (i.e., signal depletion resulting in NaN regions) into the edge extraction pipeline via the BPSW module. This fundamental shift from pure gradient-based extraction to missing-data-aware densification allows our method to extract highly dense and continuous structural contours.

## 3. Methodology

### 3.1. Overview

The essence of targetless camera–LiDAR calibration is to estimate the extrinsic parameter matrix between both sensors so that the point clouds in the LiDAR coordinate can match the corresponding pixels in images in the camera coordinate precisely. In this paper, edge features are selected as the major targets, which are randomly extracted from natural scenarios. The primary advantage of these features lies in their ubiquity across both outdoor and indoor environments. Furthermore, they exhibit strong correspondence between point clouds and images, facilitating reliable cross-modal matching. In this study, we assume that the intrinsic parameters of camera and LiDAR are well-calibrated, and the data from both sensors are collected simultaneously.

Point cloud is a set of data measured from LiDAR sensors. All the reflected points that belong to the point cloud set can be formulated as

(1) $$\left\{p_{L}=\left[x,y,z\right]\in {\mathbb{R}}^{3}|p_{L}\in \Phi_{L}\right\}$$

where $p_{L}$ denotes the LiDAR point; x,y,z represent 3D spatial coordinates; and $\Phi_{L}$ is the data set measured from LiDAR.

Images are captured by camera, and each can be seen as a 2D matrix Ψ with size U×V, where each pixel is defined as (*u*, *v*). So, the fundamental theorem for point cloud transforming to pixel-level data follows the rigid body transformation below:

(2) $$P_{C}=K\cdot \left[\begin{array}{cc} R & t\\ 0_{3\times 1} & 1 \end{array}\right]\cdot P_{L}$$

where *K* denotes the intrinsic parameter of the camera; $P_{C}={\left[\begin{array}{cc} p_{C} & 1 \end{array}\right]}^{T}$ and $P_{L}=\left[\begin{array}{cc} p_{L} & 1 \end{array}\right]$ represent the augmented matrix of *p_C_* and *p_L_* to match the dimensions; **R** and **t** are six degrees-of-freedom (6-DoF) transformation. Here, **R** indicates the 3D rotation matrix, determined by the rotation angles (yaw, pitch, roll), and **t** denotes the translation vector of three directions (*t*_x_, *t*_y_, *t*_z_). The rigid body transformation satisfies

(3) $$\Xi_{C}^{L}=\left[\begin{array}{cc} R & t\\ 0_{3\times 1} & 1 \end{array}\right]\in SE\left(3\right)$$

where $\Xi_{C}^{L}$ represents the extrinsic parameter matrix.

**Figure 2:**
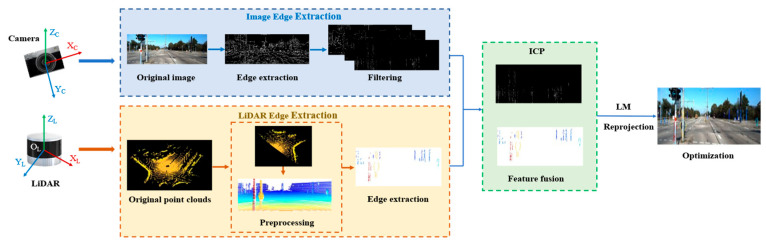
The workflow of the EdgeDenseCalib.

Figure 2 demonstrates the workflow of the EdgeDenseCalib. The pipeline consists of three parts: First, the edge features are extracted and filtered by a series of approaches from images and point clouds. Then, the point clouds are projected onto 2D frames using initial extrinsic parameters, and the ICP method is applied to search for neighboring corresponding points from both sources. Finally, a cost function is established based on distance calculation and optimized by the Levenberg–Marquardt (LM) algorithm iteratively. Details of the EdgeDenseCalib are illustrated as follows:

### 3.2. Image Edge Extraction

**Figure 3:**
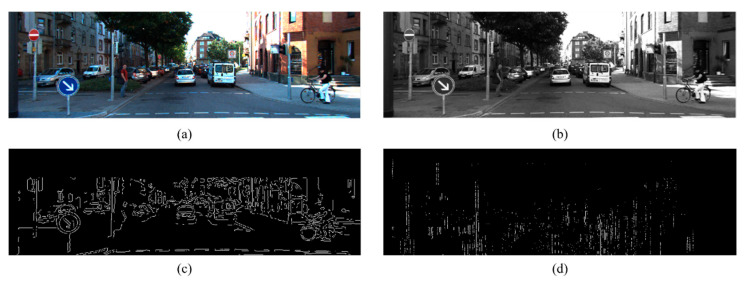
The edge feature extraction process of an image. (**a**) represents the original image of a single shot by camera; (**b**) is the grayscale of the image; (**c**) illustrates the Sobel edge extraction result with upper range cut; (**d**) shows the edge feature image after vertical and horizontal filtering.

Image edges are typically extracted depending on the gradient and orientation of the neighboring pixel values. In this paper, we adopt the classic edge extraction algorithm Canny [40] to detect edge features, as shown in Figure 3. First, the RGB images are transferred to grayscale images, and then a Gaussian filter is applied to smooth the poor edge features. We select a proper kernel size to eliminate noise from the original image. Our aim is to emphasize the most salient and distinctive edge features pertinent for calibration, while concurrently reducing the influence of noise and extraneous details to the greatest extent achievable. Then, we utilize the Sobel operator to calculate the gradient and orientation of each image pixel. The gradient and orientation value can be expressed as

(4) $$G=\sqrt{G_{x}^{2}+G_{y}^{2}}$$

(5) $$\theta =\arctan\left(\frac{G_{y}}{G_{x}}\right)$$

where G*_x_* and G*_y_* denote the horizontal and vertical component of gradient value.

Subsequently, non-maximum suppression and double-sided thresholding methods are employed to reserve the strongest edge points and filter the surrounding noise points. Moreover, we set the higher threshold 1.5 times to the lower threshold. Since LiDAR has a limited field of view (FoV) in a pitch angle of δ, we cut the upper part of the filtered edge image by turning the edge points to background to avoid unnecessary mismatching. In natural scenarios, objects with vertical features—such as poles, signs, and buildings— are more prevalent than those with horizontal features. To fully utilize these characteristics, vertical edges are treated as the primary features, while horizontal edges serve as secondary features applied to constrain the specific regions of the detected targets. Without these level constraints, there is little difference between correct $\Xi_{C}^{L}$ and incorrect alignments when matching edges from images and point clouds.

### 3.3. LiDAR Edge Processing

To achieve the alignment camera and LiDAR coordinates, we need to extract the edges from point cloud data, which are arbitrarily scattered in 3D space with distance information. In this paper, by continuously projecting the 3D LiDAR edge points onto the 2D image plane using the camera intrinsics and the current extrinsic estimates, we formulate the calibration task as a 3D–2D reprojection optimization problem. While the mathematical transformations remain in 3D–2D space, the structural alignment error is efficiently evaluated and minimized by computing the nearest-neighbor distances within the 2D image domain.

**Figure 4:**
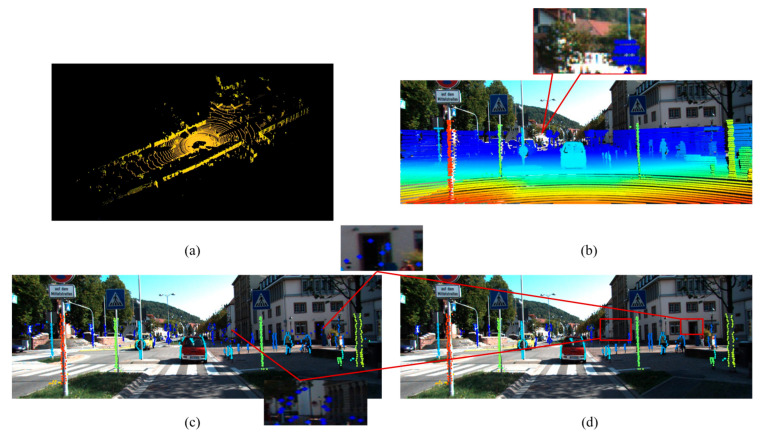
The edge feature extraction process of point cloud. (**a**) depicts the original point cloud from LiDAR; (**b**) is the projection of 3D point cloud to 2D image depending on depth; (**c**) shows the edge extraction by depth discontinuous principle; (**d**) represents the edge features after DBSCAN clustering filter.

In the LiDAR edge extraction pipeline, as shown in Figure 4, we first apply Equation (2) to complete the 3D to 2D transition and then utilize the depth information of point cloud data to build a 2D depth map.

During LiDAR scanning, distant background points often return signals of insufficient intensity compared to foreground objects. This physical limitation creates localized regions entirely devoid of point returns. An example is visible around the traffic sign in the detailed view of Figure 4b. Consequently, the CSW module alone cannot reliably detect edges adjacent to these blank regions. We therefore introduce the BPSW module to address this specific issue. The core principle of this module relies on localized missing data detection to capture the boundaries between measurable foreground objects and unreturned background areas. The algorithm evaluates the presence of missing data within this window. If the count of NaN pixels is strictly greater than zero, it indicates that the current valid point resides on the physical boundary of an object adjacent to a signal-depleted region. Consequently, this point is immediately classified as a boundary transition edge point, as shown in Figure 4c.

Finally, a depth-aware DBSCAN clustering algorithm is applied to filter the extracted edge points and effectively suppress noise. As illustrated in corresponding red boxes of Figure 4c,d, this clustering step successfully removes scattered points within the point cloud regions. The resulting edge set is cleaner and structurally more coherent, which improves the reliability of subsequent calibration.

**Figure 5:**
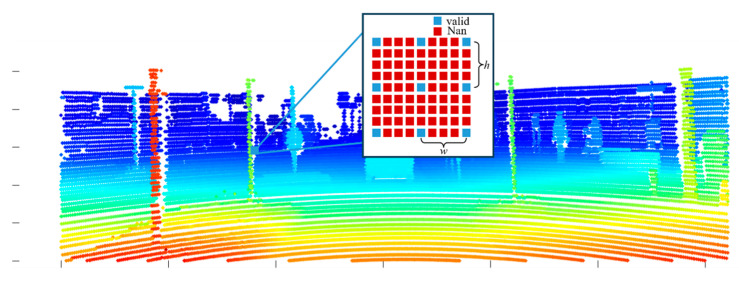
The sparse distribution of point cloud projected to the pixels, where the blue pixels represent valid values from LiDAR and the red ones are the NaN values.

Since the point clouds collected by LiDAR are sparse in space, the transformed depth map points cannot fulfill the pixels in the image size, which means the neighboring projected points exist in a gap of NaN values, as shown in Figure 5. Here, we adopt the depth discontinuous method to extract edge points of LiDAR. To achieve this goal, we design a two-stage LiDAR edge extraction mechanism which consists of a CSW module and a BPSW module. The CSW module contains a horizontal window and a vertical window. As illustrated in Figure 5, the value of the filter window width depends on the gap between continuous projected points. Based on the principle of depth discontinuity, each valid projected point is initially evaluated using the horizontal window to search for its nearest valid neighboring points. If the absolute depth difference between the projected point and any valid neighbor within this horizontal window exceeds a predefined threshold, the point is classified as an edge. Furthermore, to mitigate the risk of missing boundary features, the vertical window is subsequently applied as an enhancement step. Any point that is not identified as an edge during the horizontal search undergoes a secondary assessment within the vertical window. In this paper, the size of the horizontal window is defined as $W_{h}\times H_{h}$, where $H_{h}\in \left(h,\;2h\right)$ and *h* is 4 pixels. The size of the vertical window is defined as $W_{v}\times H_{v}$, where $W_{v}\in \left(w,\;\;2w\right)$ and *w* is 4 pixels. This dual-stage process ensures robust extraction of edge points from objects in natural scenes.

The whole process from 3D point clouds to 2D edge point set is shown in Algorithm 1.
**Algorithm 1:** LiDAR edge extraction and densification
Input: Point cloud $\Phi_{L}$

Output: The filtered projected point set $p\prime_{C}^{L}$
1Project 3D points to 2D pixel values (2)2Fill the valid pixels with depth values, the others are NaN values3for *i* = 1 to *k* do4    Use CSW module based on depth discontinuity to filter edge pixels5    Use BPSW module to enhance edge features6    DBSCAN clustering processing $p_{i}\prime_{C}^{L}$
7end

### 3.4. Optimization

Following the extraction of edge features from both images and projected point clouds, the calibration problem is formulated as a 3D-to-2D reprojection optimization task evaluated on the image plane. The core objective is to refine the extrinsic parameters by minimizing the geometric discrepancy between corresponding edge features.

To establish point-to-point correspondences, the ICP algorithm is employed. The ICP framework is highly appropriate here because the extracted edge maps from both modalities are essentially unstructured binary structures lacking explicit local feature descriptors. ICP efficiently aligns these pure geometric shapes by iteratively minimizing spatial distances without requiring complex feature representations. Given the set of filtered LiDAR edge points projected onto the 2D image plane, denoted as ${𝒫}_{L}={\left\{p_{L}^{i}\in {\mathbb{R}}^{2}\right\}}_{i=1}^{N_{L}}$ and the set of image edge points, denoted as ${𝒫}_{I}={\left\{p_{I}^{j}\in {\mathbb{R}}^{2}\right\}}_{j=1}^{N_{I}}$. For each LiDAR edge point $p_{L}^{i}$, we adopt the k-nearest neighbors (kNN) algorithm to search the corresponding points in the image edge set ${𝒫}_{I}$ based on the Euclidean distance. The kNN approach is chosen because searching for the absolute closest spatial point provides a direct, non-parametric, and computationally efficient approximation of the true structural correspondence. This establishes a set of putative correspondences $\left\{\left(p_{L}^{i},\;\;\;q_{I}^{i}\right)\right\}$, where $\;q_{I}^{i}$ is the closest image edge point to $p_{L}^{i}$.

However, due to inherent modality differences, varying fields of view, and partial occlusions, perfect one-to-one edge correspondence is practically impossible. This may lead to numerous mismatched outliers. To robustly handle these outliers and prevent them from dominating the optimization gradient, we implement a distance-based rejection strategy. Specifically, we apply a strict maximum distance threshold $D_{\max}$ during the kNN search. Any paired points with a Euclidean distance exceeding $D_{\max}$ are immediately classified as outliers and discarded.

The quality of alignment is quantified by a cost function defined as the mean squared Euclidean distances between these corresponding points:

(6) $$E\left(R,t\right)=\frac{1}{N}\sum_{i=1}^{N}{\left\|\pi \left(K\left[R|t\right]{\tilde{p}}_{L}^{3D,i}\right)-q_{I}^{i}\right\|}_{2}^{2}$$

where ${\tilde{p}}_{L}^{3D,i}$ denotes the homogeneous coordinate of the original 3D LiDAR edge point corresponding to the projected 2D point $p_{L}^{i}$. The function *π*(⋅) represents the perspective projection operation, which maps a 3D point in the camera coordinate system to its 2D pixel location.

The optimization problem is then to find the optimal rotation and translation that minimize the cost as

(7) $$R^{*},t^{*}=\arg\underset{R,t}{\min}E\left(R,t\right)$$

where $R^{*}$, $t^{*}$ denote the optimal rotation matrix and translation vector. To solve the non-linear optimization problem, we employ the LM algorithm, which is proven as an effective solution [41]. The parameter vector $\theta \in {\mathbb{R}}^{7}$ is defined as

(8) $$\theta =\left[\omega^{T},t^{T}\right]$$

where **ω** represents the quaternion vector transferred by **R**. The LM algorithm iteratively updates the parameters by solving

(9) $$\left(J^{T}J+\lambda diag\left(J^{T}J\right)\right)\Delta \theta =-J^{T}r$$

where **r** denotes the residual vector containing the distance errors for all 2D–3D correspondences. **J** is the Jacobian matrix of the residuals with respect to ***θ***. *λ* signifies a damping parameter adjusted adaptively in each iteration to ensure convergence.

## 4. Experiments

### 4.1. Dataset Preparation

To validate the EdgeDenseCalib method, several experiments are conducted on the KITTI dataset [42], which is collected data from a VelodyneHDL-64E LiDAR and a high-resolution color camera. The scanning frequency of the LiDAR is 10 Hz. The data used in the experiment is synced and rectified. The ground truth extrinsic parameters can be obtained from calibration files.

### 4.2. Qualitative Results

**Figure 6:**
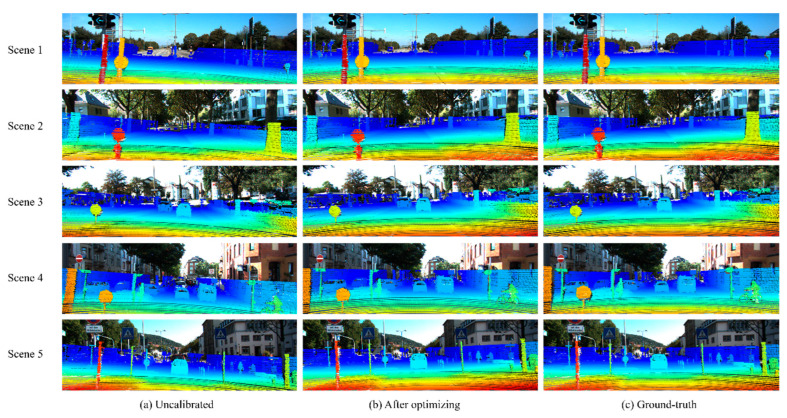
The calibration results in several scenes. (**a**) represents the uncalibrated point clouds projected on corresponding images; (**b**) depicts the point clouds after optimizing; (**c**) is the ground-truth.

To visualize the calibration results, we select five different scenes from the KITTI dataset, as illustrated in Figure 6. To assess the alignment quality across modalities, the 3D LiDAR point clouds are projected onto their corresponding image planes using the calibration parameters. The ‘Jet’ colormap is employed to encode the radial distance of each LiDAR point from the sensor origin. As shown in Figure 6a, we add a perturbation of 2° rotation (yaw, pitch, roll) and 2 cm translation (x, y, z) to the aforementioned scenes. The re-projection point cloud after optimizing and the ground-truth are displayed in Figure 6b,c. The projected points exhibit good overlap with the relevant image structures, such as poles, trunks, signs, and vehicle contours.

### 4.3. Quantitative Results

In this experiment, a perturbation of 2° rotation on X, Y, Z axes and 2 cm translation to the ground-truth are firstly applied. After optimizing, we compare the calibration error with the ground truth parameters, which are defined as the absolute value of deviation. The detailed results are shown in Table 1.

Referring to the data in Table 1, the results from Figure 6 of Scene 1 to Scene 5 are as follows: Our EdgeDenseCalib achieves a mean translation error of 0.903 cm (X, Y, Z: 0.818 cm, 1.510 cm and 0.382 cm) and a mean rotation error of 0.105° (yaw, pitch, roll: 0.064°, 0.190°, 0.062°).

Then, we conduct a comparative analysis of the EdgeDenseCalib to other methods, namely, SOIC [1], Levinson [36], Zhang [38], SE-Calib [43], EdgeCalib [8], and Multi-FEAT [10]. These methods utilize edge features, line features, and semantic edge features to search the alignment between LiDAR and camera sensors. As the methods of SOIC [1], Levinson [36], Zhang [38], and SE-Calib [43] only report calibration results on rotation parameter, we compare the accuracy of the rotation parameter. As illustrated in Table 2, EdgeDenseCalib outperforms the other aforementioned baseline methods when tested on the KITTI dataset. Compared to the edge-based methods (e.g., SOIC [1], Levinson [36], Zhang [38], SE-Calib [43], and Multi-FEAT [10]), EdgeDenseCalib achieves 33.1%, 89.9%, 79.7%, 51.2%, and 88.1% on rotation error, respectively. While EdgeCalib [8] exhibits a slight advantage of 0.019° in rotation calibration, the proposed EdgeDenseCalib outperforms the former by 8.2% in translation accuracy. Then, we compare EdgeDenseCalib to some learning-based methods, like LCCNet [34] and CFNet [35]. Admittedly, such methods outperform EdgeDenseCalib in both rotation and translation metrics. However, our purely geometric edge-based approach demonstrates higher stability and generalization capabilities in unseen environments, avoiding the domain-shift issues typical of data-driven models.

To assess the generalization ability of EdgeDenseCalib across diverse real-world scenarios, we extended the evaluation to an additional 5 scenes from the KITTI dataset. These scenes encompass a variety of environmental conditions including urban interaction, a residential street, a tree-lined street, a campus, and a city street. For each sequence, we applied an initial perturbation of 2° and 2 cm and executed the optimization pipeline. The results are shown in Table 3.

The results in Table 3 demonstrate consistent calibration accuracy across diverse environments. Rotation error remains below 0.2° in all scenes, with the lowest values observed in campus (0.063°) and on the city street (0.076°), where vertical structures are abundant. Overall, EdgeDenseCalib exhibits reliable generalization across varying real-world scenarios.

### 4.4. Ablation Analysis

In this section, a series of ablation experiments are conducted to evaluate the contribution of each key component in the EdgeDenseCalib framework.

**Impact of Vertical Edge Features.** In contrast to conventional approaches that directly employ the Canny detector for generic edge extraction, our method specifically emphasizes vertical edge features, which are ubiquitously present in natural scenes. To assess the importance of such features in calibration performance, a comparative study is performed against the baseline Canny-based edge detection method. Under identical experimental conditions on the KITTI dataset, as summarized in Table 4, the incorporation of vertical edges contributes to a 92.1% reduction in rotation error. The Canny-based method tends to converge to local optima during the matching process. This is because, without the extraction of vertical edge features, the edges detected by the Canny algorithm remain excessively dense and cluttered, even after Gaussian filtering. Such clutter introduces ambiguities that lead to incorrect point correspondences during the matching process. These results confirm the effectiveness and accuracy of the proposed feature design, demonstrating its critical role in enhancing cross-modal spatial alignment.

**Impact of CSW module.** To enhance the extraction of edge information from point cloud data, the raw point cloud is first spatially transformed from 3D to 2D through pixelization. Due to the inherent sparsity of the point cloud and the rounding operation during projection, we introduce a CSW module to obtain richer and more structurally coherent edge features. To evaluate the effectiveness of this module, two independent simulation experiments are conducted under identical conditions: one incorporating the proposed CSW module, and the other employing only a conventional horizontal scanning approach. As shown in Table 4, the results demonstrate that the method with the CSW module achieves an improvement of approximately 63.2% in rotation and 53.1% in translation accuracy, confirming its contribution to more reliable and precise cross-modal calibration.

**Impact of BPSW module.** During LiDAR scanning, distant background points often fail to return measurable reflections, leading to incomplete point clouds near foreground edges. To mitigate this issue and enrich the available edge information, the BPSW module is designed. To validate the contribution of this module, an ablation study was conducted, as summarized in Table 4. The results demonstrate that the proposed BPSW module achieves an 80.4% improvement in rotation accuracy and a 95.8% improvement in translation accuracy. Without sufficient edge constraints, the limited set of LiDAR edge points can be incorrectly associated with pixels from unrelated structures, causing the optimization to converge to suboptimal local minima.

## 5. Conclusions

This study set out to address the critical challenge of targetless extrinsic calibration between cameras and LiDARs, a fundamental prerequisite for reliable multi-sensor fusion in autonomous systems. The primary bottleneck in natural scenarios is the inherent feature-density imbalance between the sparse 3D point clouds and the fine-grained 2D images. To overcome this, we proposed EdgeDenseCalib, a novel and fully automatic calibration framework that enhances edge feature extraction and densification to establish highly robust cross-modal correspondences without requiring artificial targets or human intervention.

Our core methodological contribution lies in a meticulously designed two-stage LiDAR edge-enhancement strategy. Specifically, the CSW module is developed to capture continuous structural edges by evaluating depth discontinuities in both horizontal and vertical directions. To tackle the issue of missing point returns from distant backgrounds, the BPSW module is introduced, effectively enriching the sparse edge representation. Furthermore, an optimization method based on the ICP framework is employed to rigorously minimize the reprojection errors between the densified LiDAR edges and the image edges.

Extensive experimental evaluations on the KITTI dataset demonstrate the superior effectiveness and robustness of our approach in real-world environments. Quantitatively, EdgeDenseCalib achieves highly accurate calibration results, with a mean rotation error of 0.105° and a mean translation error of 0.903 cm. Compared to existing state-of-the-art edge-based calibration methods, our approach reduces the mean rotation error by up to 89.9%, confirming the significant advantage of our feature densification design. Ablation studies further validate that the emphasis on vertical edges and the integration of the CSW and BPSW modules are indispensable for preventing the optimization from falling into local optima.

Despite its promising performance, our method currently relies primarily on geometric edge features. In extreme environments where distinct structural edges are severely lacking (e.g., blank tunnels or vast open fields), the calibration accuracy might be compromised. For future work, we plan to improve the algorithm’s robustness in edge-deficient scenarios by incorporating semantic segmentation cues and photometric textures. Additionally, extending this framework to accommodate multi-camera systems and non-repetitive scanning solid-state LiDARs represents an important direction for our ongoing research.

## Figures and Tables

**Table 1:** Experiment results on rotation and translation.

Scene	Rotation Error (°)				Translation Error (cm)			
	Mean	Yaw	Pitch	Roll	Mean	X	Y	Z
1	0.18	0.16	0.23	0.15	0.517	0.78	0.10	0.67
2	0.073	0.01	0.20	0.01	1.093	0.45	2.50	0.33
3	0.18	0.06	0.42	0.06	1.117	1.04	2.21	0.10
4	0.073	0.07	0.07	0.08	1.573	1.79	2.18	0.75
5	0.02	0.02	0.03	0.01	0.216	0.03	0.56	0.06

**Table 2:** Comparison results with other calibration methods.

Methods	Rotation Error (°)				Translation Error (cm)			
	Mean	Yaw	Pitch	Roll	Mean	X	Y	Z
SOIC [1]	0.157	0.070	0.170	0.230	/	/	/	/
Levinson [36]	1.043	0.991	1.067	1.037	/	/	/	/
Zhang [38]	0.517	0.493	0.452	0.487	/	/	/	/
SE-Calib [43]	0.215	0.180	0.223	0.206	/	/	/	/
EdgeCalib [8]	0.086	0.124	0.036	0.097	0.977	1.168	1.256	0.507
Multi-FEAT [10]	0.879	0.688	1.204	0.745	4.933	1.6	7.3	5.9
Ours	0.105	0.064	0.190	0.062	0.903	0.818	1.510	0.382

**Table 3:** Performance on extended KITTI scenes.

Scenes Name	Environment Type	Rotation Error (°)	Translation Error (cm)
2011_09_26_drive_000012	Urban interaction	0.088	1.115
2011_09_26_drive_000180	Residential	0.092	1.324
2011_09_26_drive_000243	Tree-lined	0.196	0.998
2011_09_26_drive_000274	Campus	0.063	1.023
2011_09_26_drive_000303	City street	0.076	0.756

**Table 4:** Comparison results of ablation experiments. The “**↑**” denotes the relative rotation/translation error with respect to EdgeDenseCalib.

Setting	Relative Rotation Error (°) ↑				Relative Translation Error (cm) ↑			
	Mean	Yaw	Pitch	Roll	Mean	X	Y	Z
w/o vertical edge	1.218	1.231	1.177	1.245	18.16	21.53	3.16	29.79
w/o CSW	0.180	0.152	0.217	0.171	1.024	0.485	2.451	0.135
w/o BPSW	0.432	0.624	0.025	0.646	20.72	20.26	17.16	24.74
Ours	0.105	0.064	0.190	0.062	0.903	0.818	1.510	0.382

## Data Availability

The original contributions presented in this study are included in the article. Further inquiries can be directed to the corresponding author.
